# Vampire Bat Rabies: Ecology, Epidemiology and Control

**DOI:** 10.3390/v6051911

**Published:** 2014-04-29

**Authors:** Nicholas Johnson, Nidia Aréchiga-Ceballos, Alvaro Aguilar-Setien

**Affiliations:** 1 Animal Health and Veterinary Laboratories Agency, Woodham Lane, Surrey, KT15 3NB, UK; 2 Rabies Laboratory, Virology Department, Institute of Epidemiology Diagnostic and Reference (InDRE), Francisco de P. Miranda #177Bis. Colonia Unidad Lomas de Plateros. 01480 D.F., Mexico; E-Mail: nhyxbiogirl@gmail.com; 3 Medical Immunology Research Unit, Paediatric Hospital, Naional Medical Center “Siglo XXL”, Mexican Social Security Institute (IMSS), Av. Cuauhtémoc 330, Col. Doctores, 06720, D.F., Mexico; E-Mail: estiviro@hotmail.com

**Keywords:** vampire bat, *Desmodus rotundus*, rabies virus, transmission

## Abstract

Extensive surveillance in bat populations in response to recent emerging diseases has revealed that this group of mammals acts as a reservoir for a large range of viruses. However, the oldest known association between a zoonotic virus and a bat is that between rabies virus and the vampire bat. Vampire bats are only found in Latin America and their unique method of obtaining nutrition, blood-feeding or haematophagy, has only evolved in the New World. The adaptations that enable blood-feeding also make the vampire bat highly effective at transmitting rabies virus. Whether the virus was present in pre-Columbian America or was introduced is much disputed, however, the introduction of Old World livestock and associated landscape modification, which continues to the present day, has enabled vampire bat populations to increase. This in turn has provided the conditions for rabies re-emergence to threaten both livestock and human populations as vampire bats target large mammals. This review considers the ecology of the vampire bat that make it such an efficient vector for rabies, the current status of vampire-transmitted rabies and the future prospects for spread by this virus and its control.

## 1. Introduction

Rabies is a zoonotic disease caused by viruses belonging to the genus *Lyssavirus*; family *Rhabdoviridae*. It is now considered a re-emerging disease in different countries of the world and is associated with increased rates of reservoir contact [1].The disease is transmitted by the bite of a rabid animal; usually dogs; although bats act as a reservoir for lyssaviruses in many regions of the world [2]. 

Through concerted action across the Americas, rabies virus transmission from dogs to humans has been controlled in virtually all countries of the region. This is reflected in the continued decline in human rabies over the past two decades [3]. However, rabies virus is found in many species of bat in the Americas and there continue to be cases of transmission from vampire bats to humans and livestock [4,5,6].

The earliest description of human death associated with vampire bat attacks comes from the time of the Spanish conquest of the Americas in the 16th century [7]. Rabies virus transmission from vampire bats to cattle has been recognized for over one hundred years [8,9] and continues to be a major burden to the livestock industry [10]. The first documented outbreak of human rabies of vampire bat origin occurred in Trinidad in 1927 [11,12]. These outbreaks have continued to the present day and are a challenge to both the veterinary and human health agencies, which face sudden outbreaks of rabies in livestock or human populations. Such events often occur in remote regions where access to healthcare is restricted [13].

Vampire bats preferentially prey on livestock [14]. Livestock and horses are generally larger than indigenous wildlife prey species, are more abundant and tend to stay in the same location for extended periods. Once a colony of vampire bats has located a herd of animals, they are then able to return to the same herd on subsequent nights. This is particularly true for cattle. The introduction of cattle, and other livestock species, during the post-Columbian conquest provided the vampire bat with an abundant food supply [15]. Humans have also provided vampire bats with roosting sites in the form of buildings, bridges and wells. This in turn has contributed to an increase in the number and size of vampire bat colonies, and enlarged the population that can act as a reservoir for rabies virus. Deforestation, a consequence of land clearance for logging and modification for agriculture has simultaneously reduced the numbers of natural prey species and brought vampire bats into contact with livestock and man.

Bovine rabies in Latin America is commonly called derriengue, a Spanish word for a fatal paralytic disease [16]. The infected animals exhibit signs of restlessness or excitement with sudden onset of hind limb paralysis. This progresses to the fore limbs. Overt salivation is commonly observed but is believed to be due to difficulties in swallowing rather than excess saliva production. Emaciation in animals that survive for any length of time is observed but the disease is invariably fatal. 

The increase in the abundance of species such as vampire bats in the Americas has had a direct impact on human and animal health [17,18]. Moreover, regional and global change could lead to changes in vampire bat behavior and distribution that could increase the incidence of human rabies in Latin America and the potential to spread north to the United States of America. Therefore it is timely to review the impact of this disease-reservoir interaction and assess its future development.

## 2. Vampire Bat Biology and Ecology

**Figure 1:**
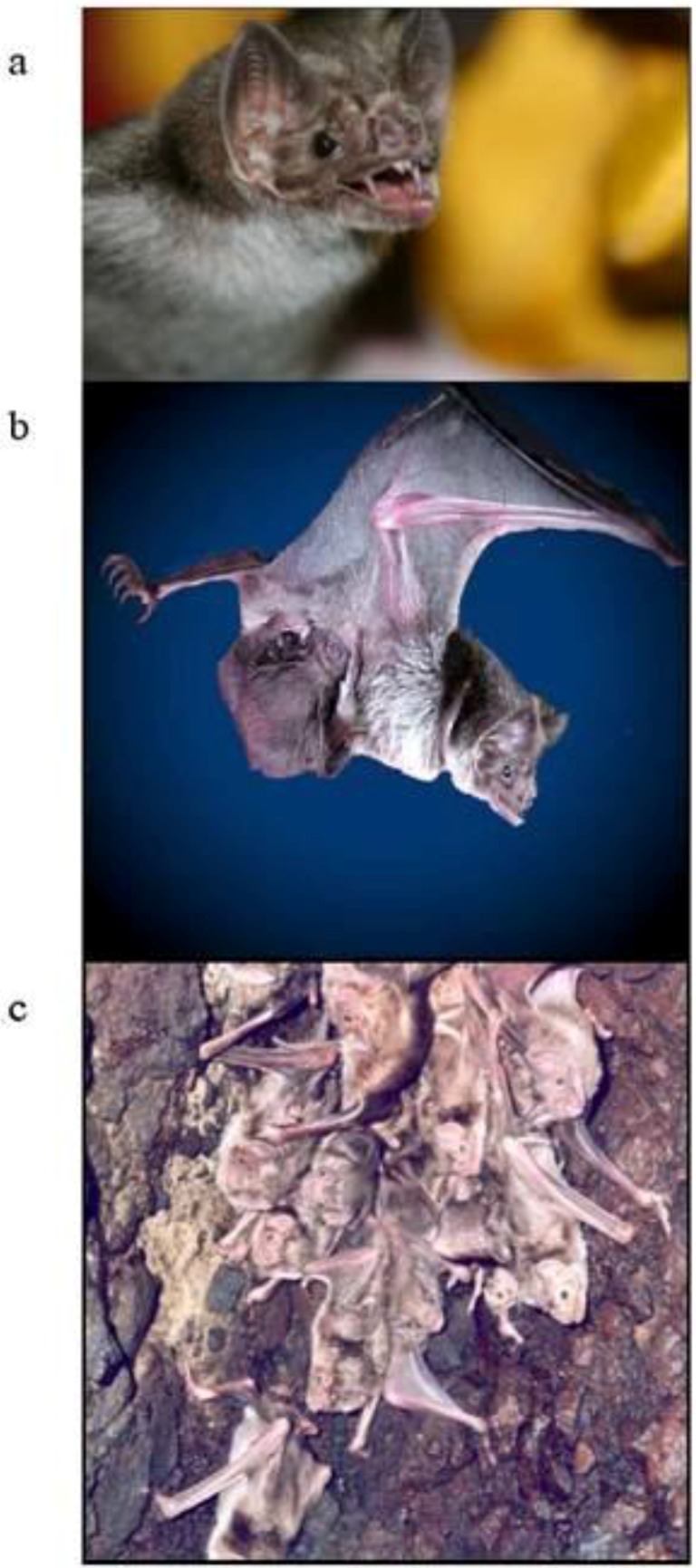
Images of the common vampire bat *Desmodus rotundus*. (**a**) A close up showing the sharp incisors used to puncture the skin of prey animals; (**b**) a female with young in flight; (**c**) a small colony of *D. rotundus*.

There are three species of blood-feeding or hematophagous bats found exclusively in Latin America (Table 1). Only one of these, the common vampire bat *Desmodus rotundus* (Figure 1), is a well known reservoir for rabies.

**Table 1:** The blood feeding bats of Latin America. All belong to the family *Phyllostomi-dae* or New-World leaf-nosed bats.

Species	Common name	Description
*Desmodus rotundus*	Common vampire bat	Weight: 30–40 g
		Wingspan: 35–40 cm
		Colony size: 20–1000
		Prey: mammals
*Diaemus youngi*	White-winged vampire bat	Weight: 30–45 g
		Wingspan: 32–35 cm
		Colony size: up to 30
		Prey: birds
*Diphylla ecaudata*	Hairy-legged vampire bat	Weight: 25–40 g
		Wingspan: 37–45 cm
		Colony size: 20–500
		Prey: mammals and birds

Vampire bats feed at night and prefer moonless nights to avoid detection by prey animals. The common vampire bat has a number of adaptations for blood-feeding that enhance its ability to transmit rabies virus. Firstly, the species feeds on a wide range of hosts, including humans, although will preferentially feed on large livestock such as cattle and horses. The teeth of *D. rotundus* are blade-like (Figure 1a) and undergo thegosis, a process of self-sharpening in which the upper incisors brush against the lower canines [19]. This ensures that the bat can deliver a virtually painless bite, creating a distinctive crater-like wound on the host. The wound is sufficiently deep to induce profuse bleeding. Clotting is prevented by secretion of anticoagulant in saliva that is channeled down a groove on the dorsal surface of the tongue. Finally, the species forms colonies (Figure 1c), occasionally consisting of thousands of animals [20].

**Figure 2:**
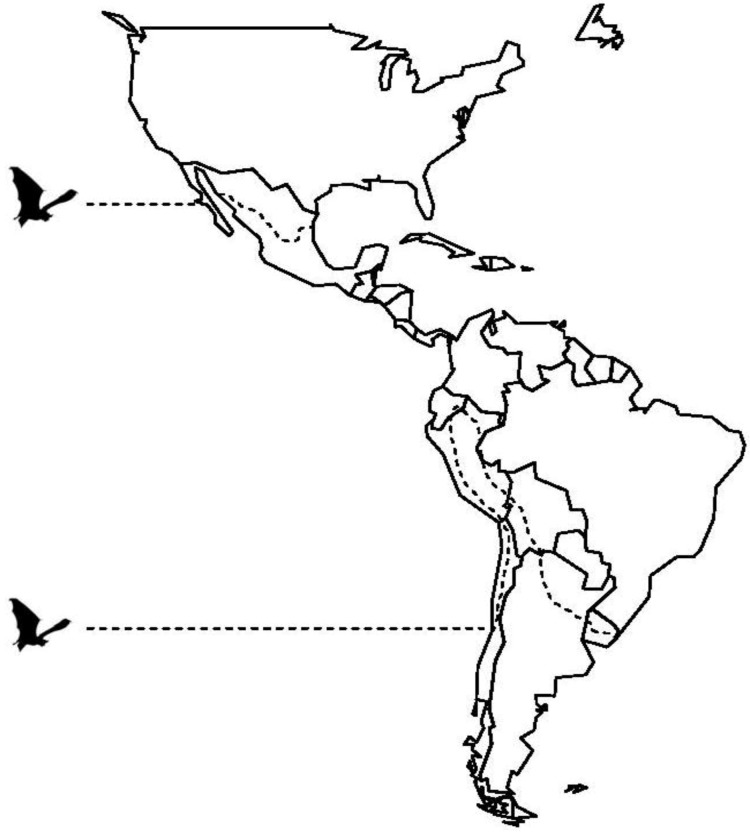
Limits of distribution (dashed line) for all vampire bat species in Latin America.

The common vampire bat is found from Mexico to northern Argentina (Figure 2) and instances of vampire bat-transmitted rabies mirror this distribution but with particular foci in Mexico and the Amazon Basin.

## 3. Rabies Virus Dynamics in Vampire Bat Populations

Several studies describing the epidemiology of rabies virus within populations of vampire bats suggest that the virus infects many individuals; some die and others survive exposure, demonstrable by the presence of antibody against the virus. The disease disappears from the bat population in time and does not return until a sufficient number of susceptible bats have re-entered the population [21,22,23]. A recent study combined experimental and field observations to describe a model in which rabies virus is maintained within the populations of vampire bats in Peru [24]. They concluded that the probability of developing a lethal infection upon exposure to rabies is quite low for vampire bats (~10%), and it is more likely that most exposures are subclinical and immunizing. This enables long term viral persistence in colonies of a species with a slow reproductive rate by a high frequency of immunizing exposures. The authors also highlighted the role of immigration in the dynamics of rabies virus within the vampire populations as the method of virus spread that leads to sporadic but lethal vampire bat rabies virus infection. Frequent interactions among bats from different colonies are necessary to maintain the chain of transmission. Similarly, high seroprevalence to rabies virus in other bat species suggests that frequent survival after exposure may be a general feature of bat rabies, nevertheless incubation and infectious periods remain an important unknown.

There is strong evidence that rabies in the vampire bat moves in wave-like patterns through regions. This assertion is based on detailed studies of rabies outbreaks in Argentina [22]. Ahead of the wavefront, there is low seroprevalance and an absence of detectable virus [25]. The wavefront is characterized by detection of virus in vampire bats but with a low seroprevalence. As the wavefront moves on, the presence of virus declines and the remaining bats are predominantly seropositive bats. This type of “migratory epidemic” often follows the course of rivers because of the greater numbers of roosting sites and access to water that is necessary for cattle ranching. Retrospective analysis of the outbreak of rabies in Trinidad that began in 1923 has provided supportive evidence for this [26]. The epidemic began in cattle in the north east of the island. This is the closest point to Venezuela on the South American mainland and easily crossed by flying animals. The outbreak spread to the south east by 1929. Then, for reasons that are not fully explained, cases in humans began in the same year. Incidence of human rabies cases suggested a northwards spread of the epidemic. Analysis of the case distribution and land use revealed an association with plantations that grew crops such as cocoa, coffee, banana and citrus fruits. Both examples are from regions on the edge of vampire bat distribution. Less structured temporal-special patterns of persistence could also occur and a recent longitudinal serology study suggests that rabies virus, once introduced into a bat population can persist for a number of years through interactions between colonies [13]. Virus naïve juvenile bats appear to play a critical role in virus persistence. Vampire bat bites lead to rabies transmission although the observation of seropositive humans in areas of vampire bat activity without vaccination history against rabies virus suggests that biting, in some cases, does expose humans to sufficient virus to stimulate antibody production without progression to disease [27].

Rabies in vampire bats has been investigated through experimental infection. Moreno and Baer [28] inoculated captive *D. rotundus* bats with a vampire bat-derived virus by the intracranial (IC), intramuscular (IM) and sub-cutaneous (SC) routes. Disease development was dependent on inoculation route and virus dose with IC inoculation inducing rabies in all recipients, whereas only a high virus dose (>562 mouse intracerebral lethal dose _50_) caused disease in the majority of bats inoculated by other routes. Virus was detected in both salivary glands and saliva from bats that developed rabies. Later studies have provided descriptions of rabies in vampire bats. The initial sign in captive bats is a decrease in blood consumption [29] leading to dehydration. No aggression was observed although neurological signs included wing paralysis, tremors and difficulty in walking [29,30]. RABV distribution in naturally infected bats is predominantly in neurological tissue followed by the tongue, associated with the presence of salivary glands [31]. This is similar to the virus distribution observed for related lyssaviruses in European insectivorous bats [32]. There appears to be little difference between the pathology of rabies infection in vampire bats and other mammals.

## 4. Virus Typing

The rabies virus genome is a negative-sense, single-stranded RNA genome approximately 12,000 base pairs in length. This contains five discrete coding sequences that encode the structural proteins, nucleoprotein, matrix and glycoprotein, and non-structural proteins, phosphoprotein and RNA-dependent polymerase. All lyssaviruses contain this simple genomic structure [33]. The main benefit of virus typing methods for investigating vampire bat rabies is the identification of the source of infection in humans and livestock in the absence of records of either a bat bite or vampire bat activity in a particular area.

### 4.1. Antigenic Typing

Until the introduction of virus typing by monoclonal antibodies, it was impossible to distinguish between rabies viruses isolated from infected brain material. Typing, using this method, allows the differentiation of viruses based on the binding, or not, of panels of monoclonal antibodies directed against the virus nucleoprotein [34] or glycoprotein. This approach has been applied to rabies viruses found in the Americas initially to discriminate between those viruses found in different sylvatic species including bats [35] and to discriminate virus variants of urban or sylvatic infections. Such an approach continues to detect new variants of rabies virus of sylvatic origin [36]. In studies conducted in New World rabies viruses, isolates of vampire bat origin are classified within Antigenic variants(AgV-) 3 and 11 [37].

### 4.2. Phylogenetic Typing

**Figure 3:**
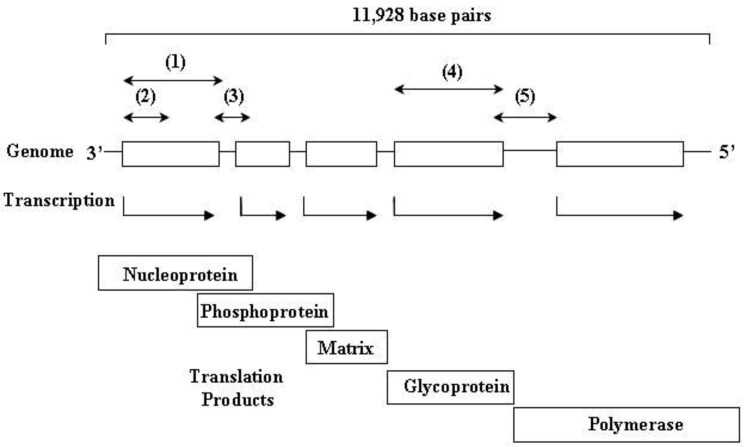
Schematic of the rabies virus genome showing transcription of the five genes to form the nucleoprotein, phosphoprotein, matrix, glycoprotein and polymerase or L gene. Sections of the genome that are commonly used for phylogenetic analysis of rabies viruses are indicated: (1) Complete nucleoprotein coding sequence; (2) partial nucleoprotein coding sequence; (3) nucleoprotein-phosphoprotein intergenic region; (4) complete glycoprotein coding sequence; (5) glycoprotein-L intergenic region.

Comparison of genomic sequences in order to discriminate rabies viruses began in the early 1990s [38] and has expanded dramatically to infer virus variation across time and space. Sequence comparison to generate phylogenies, often represented in the form of a phylogenetic tree, has enabled fine discrimination of rabies viruses. Molecular epidemiology of rabies virus has transformed the understanding of the virus and its relationship to a range of reservoirs including the spread of fox rabies [39], dog rabies [40] and bat rabies [13]. Different fragments of the RABV genome have been used for phylogenetic analysis (Figure 3). Most studies focus on a partial fragment [41] or the complete nucleoprotein coding sequence [42]. Other sequences selected include the phosphoprotein gene [37] or the glycoprotein-L protein intergenic region [43]. There is no consensus on the most appropriate fragment of the rabies virus genome to use and different regions of the genome appear to give similar results [44].

The ability to associate a particular virus lineage with a particular reservoir host has greatly assisted in revealing the interrelationships between rabies virus and New World bats. Analysis of RABVs in North American insectivorous bats indicate that particular lineages are associated with particular bat species, possibly through adaptation to the host, and that transmission between species is rare or rarely detected [45]. However, the feeding method of vampires greatly increases the opportunity for cross-species transmission of virus. Vampire and frugivorous bats were observed to utilize the same roost locations in Mexico [46] so it is highly likely that transmission events between the two could occur and indeed rabies transmission between vampire and frugivorous bats have frequently been reported [47,48,49]. In Argentina rabies viruses isolated from frugivorous bats (*Artibeus lituratus*) were identified to be a variant associated with hematophagous bats through the use of monoclonal antibody panels. In Brazil it has been demonstrated that rabies viruses isolated from Brazilian frugivorous bats (*Artibeus* spp.) were phylogenetically characterized as the vampire-bat related RABV [48]. Also in Brazil, Albas and co-workers [50] found that amongst rabid bats, AgV-3, normally associated with populations of *D. rotundus*, was found in 71.4% (15/21) of non-haematophagus bat species sampled. AgV-3 was also identified in 90.9% (10/11) of the frugivorous bats *Artibeus lituratus* and 50% of the insectivorous bats (one *Lasiurus blossevillii*, one *Molossus molossus*, one *Eptesicus furinalis*, and three *Lasiurus ega*), and the nectivorous Pallas’s long-tongued bat (*Glossophaga soricina*). The genetic variant related to haematophagous bats was identified in 75.8% (22/29) of the studied samples. Those results were also observed in studies with samples isolated from bats from the state of São Paulo and other Brazilian regions. An intensive investigation of vampire bat rabies in Peru and neighboring countries identified four discrete lineages [42]. In addition to genetic variation, there was also a degree of geographical separation although some lineages appear to occupy the same areas and this may reflect the presence of multiple infected colonies. The evidence suggests that vampire bats are highly effective at transmitting RABV to other bat species although an alternative interpretation might be that the AgV-3 is not entirely specific to vampire bats and could be transmitted within non-vampire bat species.

## 5. Impact on Livestock

Fossil vampire bats have been detected throughout the Americas dating from the late Pleistocene age over two million years ago. Images of what are clearly vampire bats were created by the Mayan civilization and descriptions of vampire bats were recorded by Spanish explorers in the 16th century [7]. It has been conjectured that the European conquest of the New World had a major impact on the vampire bat due to the introduction of livestock, which proved a highly accessible target for vampire predation. Vampire bats show a preference for livestock, particularly larger animals such as cattle, horse and sheep because they tend to remain inactive and stationary at night, in contrast to indigenous wildlife. Also livestock herds tend to remain in the same location and vampires are capable of relocating the same site over many nights. The direct effect of vampire bat predation is to weaken individual animals, especially juveniles, if the animal is subject to repeated attacks over a short period. This in turn can lead to increasing susceptibility to other diseases. Wounds can be attacked by screw-worm flies (*Cochliomyia hominivorax*) that in extreme cases can lead to death [51].

Bats have been associated with a large range of zoonotic viruses [52]. However, the most severe disease transmitted to livestock by vampire bats is rabies. This is a persistent problem throughout Latin America. Table 2 shows that the major burden of bovine rabies due to vampire bats is found in Brazil although the numbers from 2002 are considerably lower at 1321 than the 5900 recorded in 1982. This high number of rabies cases from Brazil reflects the large numbers of livestock in the country. Total bovine rabies cases peaked in 1983 at 7959 but reduced dramatically to below 1000 cases in 1989.

Despite this apparent picture of decline in rabies cases, in certain countries there is evidence that cases have increased over this twenty year period. For example in Mexico, numbers of rabies cases fluctuate widely from year to year with no clear trend in the prevalence of disease (Table 3). Many of these cases, particularly those in cattle, will be of vampire bat origin.

**Table 2:** Bovine rabies in Latin America in 1982 and 2002 (data from the Pan American Health Organisation).

Country	1982	2002
Argentina	92	13
Bolivia	159	59
Brazil	5900	1321
Chile	0	0
Colombia	139	47
Ecuador	45	16
El Salvador	7	19
Guatemala	22	11
Honduras	19	0
Mexico	35	154
Nicaragua	1	2
Panama	8	9
Paraguay	9	79
Peru	32	110
Suriname	0	5
Venezuela	54	19

**Table 3:** Rabies cases reported in Mexico between 2003–2011 from “Rabies surveillance in the United States during 2003–2011”. The source references are included in the year column.

Year	Human	Dogs	Cattle	Bats	Other	Total
2003 [53]	1	75	201 (60.7)	13	41	331
2004 [54]	3	45	186 (69.7	0	33	267
2005 [55]	8	103	252 (63.8)	10	22	395
2006 [56]	9	77	181 (62.0)	5	20	292
2007 [57]	0	42	227 (78.8)	0	19	288
2008 [58]	3	31	183 (77.9)	0	18	235
2009 [59]	4	12	134 (76.6)	0	25	175
2010 [60]	4	20	296 (82.0)	0	41	361
2011 [61]	3	20	121 (80.1)	0	7	151

## 6. Impact on Public Health

Historically, human mortality due to rabies transmitted by vampire bats has remained low because bats do not usually attack humans. However, prior to the 1970s, there had been 150 human deaths reported that were attributed to transmission by vampire bat attacks [21]. Aggression, in the form of blood feeding by *D. rotundus*, is currently the main cause of human rabies in Brazil [50]. In the absence of livestock, humans can become victims of vampire attacks, particularly if sleeping outdoors or in buildings to which bats can gain access. Buildings occupied by indigenous peoples, or those who make temporary visits to the Amazon jungle such as loggers and miners, are often temporary structures and provide no barriers to vampire bat entry. Bites are to exposed areas of the skin such as toes and the face. In 2013, four out of nine human rabies cases reported to the Pan-American Health Organization were transmitted by hematophagous bats [62]. A recent human case of rabies resulting from the bite of a vampire bat was reported from the US state of Louisiana in a migrant worker from Mexico ([63] and Box 1).

The number of rabies cases associated with wild species, especially those transmitted by vampire bats, seems to be increasing and the patterns of occurrence of this disease are also changing [36,64,65]. This may be due to four main causes, two (1 & 2) associated with increased reporting and diagnosis and two (3 & 4) associated with changes to vampire bats demography and distribution: (1) an increase in cases may be due to improved disease reporting—the frequency with which the inhabitants of rural areas reported cases has increased but it could indicate that the incidence of rabies has not changed and only the ability to compile these reports has been improved; (2) diagnostic techniques have progressed in viral identification and typing, allowing identification of the true reservoir host of a rabies virus; (3) it is possible that vampire bats, which besides dogs, are the most frequently reported source of human rabies, are increasing and expanding their populations due to augmented habitat fragmentation caused by changes in the land use and farming in the tropics and incurring a higher frequency of contact between human and reservoirs [66]; (4) climate changes are playing a major role, more than previously considered, in the distribution and abundance of reservoir species and the frequency of outbreaks, this factor may be dominant if it is considered that vampire bats are restricted by low temperatures in their environment and shelter [67]. Each of these, either individually or in combination, could have increased the reported number of human infections with rabies virus of vampire bat origin. Additionally, we have witnessed the presence of rabies variants proceeding from vampire bats in cattle located over 2800 meters above sea level, in places where the disease was not recorded previously, e.g., Tutotepec municipality in Hidalgo State of Mexico, confirming the progress of the disease [68].

**Box 1. Case History: An imported case of human rabies in Louisiana, USA, due to a vampire bat bite.**

In July, 2010, a 19 year old male migrant worker from Mexico developed fatigue with pain in his left hand and shoulder. He sought medical attention on July 30th and was transferred to New Orleans on August 3rd when his symptoms persisted. Further physical examination revealed generalized areflexia and a drooping left upper eyelid. A cerebrospinal fluid (CSF) sample contained elevated white blood cells (8 cells/mm^3^) but glucose levels were normal. The patient developed a fever with a temperature of 38.4 °C and he became generally less responsive. On August 20th, rabies virus-specific IgM and IgG were detected in the patient’s CSF and serum. On August 21st, after discussion with the patient’s family, ventilator support was removed that the patient died shortly after. Rabies virus was confirmed by detection of viral antigen in brain tissue taken at postmortem. A link to vampire bats was suggested by antigenic typing of the virus isolated from the patient and genetic analysis of nucleic acid sequencing derived from reverse transcription polymerase chain reaction.

A public health investigation was conducted that involved interviewing the patient’s family. The patient was originally resident in Michoacán, Mexico, where vampire bats were know to be present locally. Through an interview with the patient’s mother it transpired that he had been bitten by a vampire bat on the left heel whilst sleeping in the family residence. This was reported to have occurred on July 15th. The patient did not seek any medical attention for the bite and there was no evidence of vaccination for rabies.

## 7. Control of Vampire Bats and Vampire Bat Rabies

The observation of vampire rabies movement suggests that a landscape is covered in a network of interacting bat colonies, which include maternity roosts and satellite male roosts. Interactions are frequent, often daily, and can include behavior such as social grooming and blood-meal sharing [69]. This enables the rapid dissemination of rabies to a population by an infected individual. This knowledge has led to the implementation of rabies control based on destruction of vampire bats. Fornes and coworkers [70] described a migratory epidemic in north-east Argentina that was first reported in 1959 and moved at an average rate of 40 km per year in a southwesterly direction. The wavefront increased in length over time. In an attempt to halt this, the authors identified a 1500 km^2^ control zone where vampire roosts were located almost exclusively in wells. These wells were sealed and the bats inside destroyed by cyanide gas. One hundred and sixty nine wells were found of which 45 were occupied. One hundred and twenty eight wells were gassed and 363 bats were known to have been killed. Testing of 208 carcasses identified two as positive for rabies. A subsequent census one month later confirmed a dramatic reduction (>95%) in the vampire bat population. Further surveillance did not detect any rabies cases in cattle within the control area but some cases were reported to the south east of this zone bringing into question the long-term efficacy of this strategy when dealing with a highly mobile host species.

The impact of vampire predation on livestock is such that a number of control methods have been used. The most dramatic and destructive is that of colony destruction that in extreme cases has included the dynamiting of caves [71] or gassing with cyanide as described above. However, this approach is indiscriminate and kills other bat species. Furthermore, other occupants of the cave system are destroyed. It can also lead to dispersal of colonies if not carried out effectively, and may lead to the movement of the problem to another area. More recent studies suggest a critical role for immigration between bat colonies indicating that current culling practices, often reacting to outbreaks in livestock, when haphazardly implemented are unlikely to eliminate rabies virus. Whilst programs targeting specific colonies may limit local spillover from bats to humans or domestic animals, culling could have the opposite of the intended effect on rabies transmission by means of increasing the movement due to freeing up space or disturbance-mediated dispersal [24].

More targeted methods of control include the capture of vampire bats and coating the animal with Vaseline containing an anticoagulant such as Warfarin. The bat is then released and through mutual grooming spreads the anticoagulant throughout the colony leading to the death of its members. A disadvantage of this approach is that often bat species other than *D. rotundus* are treated and destroyed. A variation of this approach is to coat the walls of roosts with anticoagulant although this again can lead to indiscriminant killing of other bat species and the anticoagulant can persist in the environment for years. An alternative is to inject cattle with low levels of anticoagulant that has no effect on livestock and does not affect the suitability of meat for the food chain but is of sufficient strength to be lethal to vampire bats. A variation of this approach is to apply the anticoagulant to fresh wounds in a paste formulation, relying on the behavior of the vampire bats to return to a prior prey location. This approach is more costly although it is specifically targeted at vampire bats. If protection of rabies is the main concern then vaccination of cattle is effective but again is costly and not always adopted by farmers.

**Figure 4:**
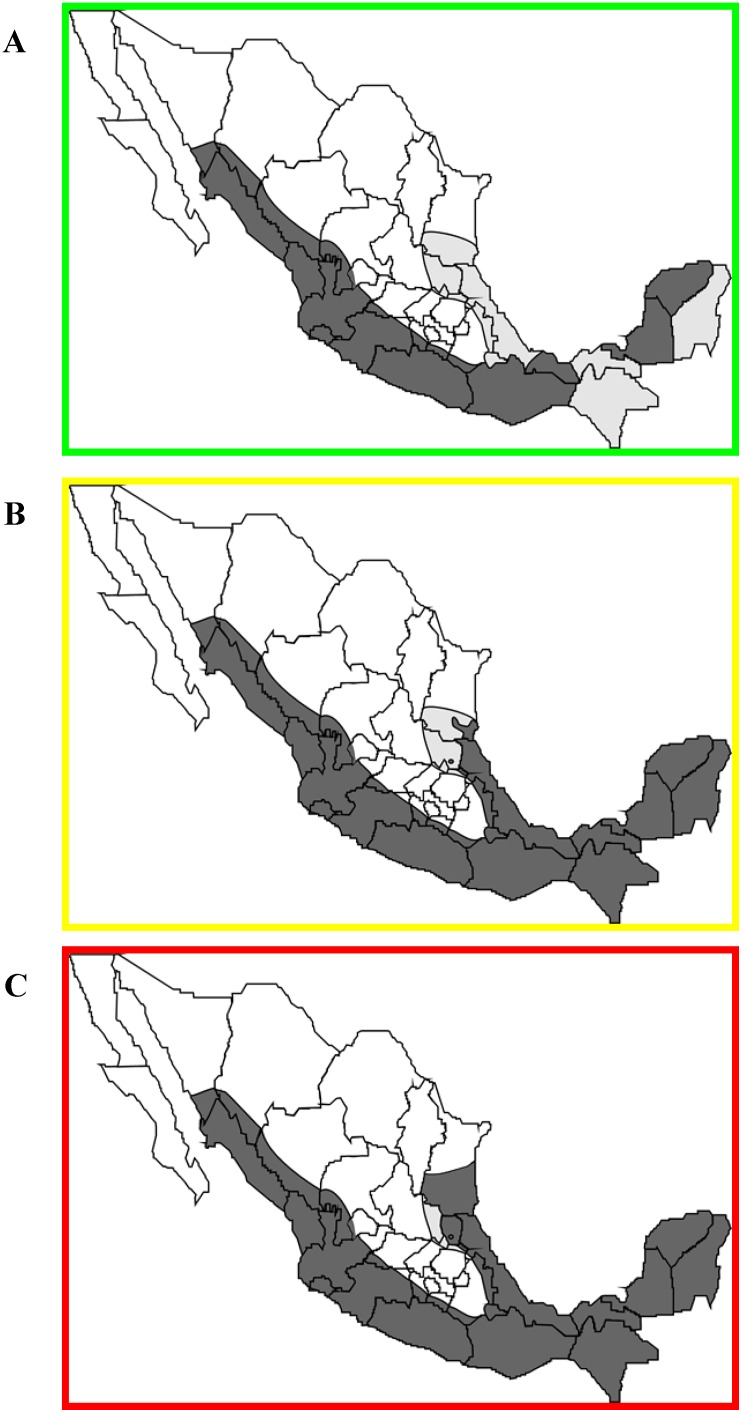
Distribution of rabies-affected areas ofMexico over three decades; (**A**) 1970s; (**B**) 1990s; (**C**) 2000s. Black indicates areas with populations of rabies-affected populations of vampire bats, grey indicatesareas with populations ofvampire bats but rabies free.

Anticoagulants have been used in most Latin American countries since the seventies. In Mexico, its use is indicated in the national recommendations in response to a disease outbreak. However, despite its application for more than 40 years, rabies in vampire bat populations has increased over time. From the 1970s rabies was present in approximately 65% of the area inhabited by the common vampire bat, and currently in the 21st century, almost 100% of this area shows bovine paralytic rabies (Figure 4, data collected from the Servicio Nacional de Sanidad, Inocuidad y Calidad Agroalimentaria, Mexico).

Streicker and co-workers [13] reported that culling campaigns in Peru over a three year period failed to reduce rabies virus seroprevalence and were perhaps counterproductive for disease control. This could imply that juvenile and sub-adult bats are more important for disease transmission than adults as these were targeted for removal. They also demonstrated that exposure to RABV was ubiquitous across geographically widespread vampire bat populations, and was at best only weakly related to bat colony size, and tended to increase following sporadic culling. This suggests that to control vampire bat populations, by capturing and treating bats with anticoagulants, only provides a temporary respite from disease transmission and that the disease rapidly returns. More studies on vampire bat population dynamics and the application of new technologies for population control e.g., reproductive control using phytoestrogens proposed by Serrano *et al.* [72] should be considered as an alternative to successful control of the disease. From a wider ecological perspective, when bats that die from anticoagulant treatment become prey to other carnivores or scavengers, this can then cause secondary death of wild fauna [73].

Experimental vaccination against rabies of vampire bats using a range of applications including both parenteral and oral administration has been attempted and shown to be effective [74,75]. However, this is unlikely to be introduced due to cost and the practical problems of locating and treating vampire bat colonies.

## 8. Conclusions

Reports of both livestock and human cases of rabies are increasing, mainly due to the continuing encroachment of human populations into areas occupied by vampire bats and there is now strong evidence that the range of vampire bats is also increasing. It is possible that vampire bats could move further north and into the United States, bringing with them the risk of rabies virus transmission to humans and livestock. Fossils of vampire bats have been found in a number of US states from warmer periods in earth history [76]. This suggests that possible effects of climate change that lead to increases in temperature could enable *D. rotundus* to move north and introduce another rabies variant into US wildlife [77]. In order to improve control of vampire rabies, Blackwood and colleagues [24] have suggested that the efforts aimed at reducing spillover through viral elimination must likely be spatially coordinated with a view to defining and synchronizing transmission dynamics within enzootic regions. Whilst control methods such as habitat destruction and indiscriminant use of anticoagulants have been used for decades, there is little evidence that they have achieved anything other than short-term respite in limited areas. Rabies in vampire bats persists and new strategies are needed that succeed in reducing the incidence of disease transmission without further destruction of bats.
